# Bioabsorbable Screws and Tibial Crest Autograft for the Treatment of Posterior Shoulder Fracture-Dislocation: A Case Report

**DOI:** 10.7759/cureus.94655

**Published:** 2025-10-15

**Authors:** Angelo De Carli, Antonio Vadalà, Benedetto Carta, Francesco Suraci, Elisa Lamberti, Cristiano Benelli, Carola Morini, Nicola Maffulli

**Affiliations:** 1 Orthopedics and Traumatology, II School of Medicine, Sant’Andrea Hospital, Kirk Kilgour Sports Injury Center, University of Rome “La Sapienza”, Rome, ITA

**Keywords:** autologous bone graft, bioabsorbable screws, proximal humerus, shoulder neck fractures, shoulder posterior dislocation

## Abstract

Posterior shoulder fracture-dislocations represent a rare and commonly misdiagnosed injury, especially in young adults. Management is controversial, with internal fixation preferred over arthroplasty in cases where humeral head viability can be preserved. This report describes a novel approach using bioabsorbable screws and tibial crest autograft for anatomical reconstruction. A 34-year-old male presented with a posterior dislocation and anatomical neck fracture of the left humerus following a motorcycle accident. Closed reduction was unsuccessful. The patient underwent open reduction and internal fixation via a deltopectoral approach. The humeral head was temporarily explanted, a tibial crest autograft was harvested and inserted, and fixation was achieved using three bioabsorbable poly-L-lactic acid (PLLA) interference screws. The postoperative course was uneventful. At three months, a full range of motion was achieved with radiological evidence of graft integration. At six months, bone healing was complete without signs of avascular necrosis. The patient returned to full activity by nine months and remained asymptomatic at the three-year follow-up. This report supports the use of bioabsorbable screws and autologous grafting as a viable option for treating young patients with posterior shoulder fracture-dislocation. The technique offers anatomical restoration while preserving future surgical options.

## Introduction

Proximal humeral fractures (PHFs) account for almost 6% of all fractures [1]. These fractures represent the third most common osteoporotic fracture, following distal radius and vertebral fractures [2]. PHFs are most common in patients over 65 years of age, with a 2:1 female to male ratio, typically following a fall from standing height [3]. In younger individuals, particularly males, these fractures are primarily caused by high-energy trauma, such as falls from height or motor vehicle accidents [3].

Most PHFs are managed nonoperatively with good functional outcome as they are minimally displaced [4]. However, about 15-20% of them are displaced and may require surgery [5]. Available surgical treatments include closed reduction and percutaneous pinning, open reduction and internal fixation (ORIF) with locking plate and screws, hemiarthroplasty, total joint arthroplasty, and reverse total shoulder arthroplasty [6]. The treatment must be patient-specific, considering fracture pattern, age, comorbidities, vascularization, functional requirements, and rotator cuff integrity [6].

We report a case of a 34-year-old patient with an anatomical humeral neck fracture and humeral head posterior dislocation treated with bioabsorbable screws and autologous bone graft from the iliac crest. Informed consent was obtained from the patient for publication of the details of this case.

## Case presentation

**Figure 1:**
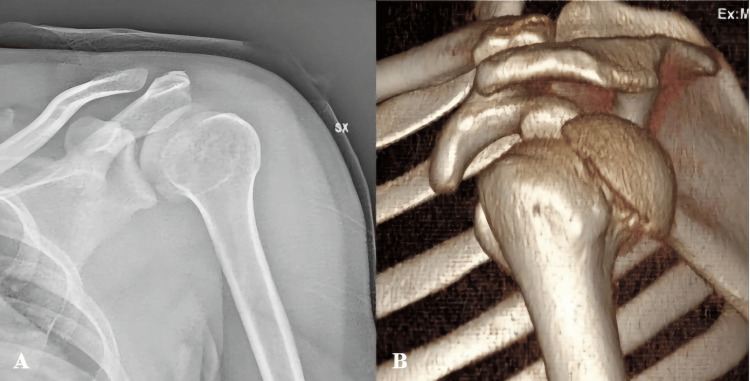
(A) Initial plain radiograph and (B) CT scan 3D reconstruction of the affected shoulder.

A 34-year-old right-hand-dominant man sustained a motorcycle accident. He presented to the Accident and Emergency Department with swelling, tenderness, severe pain, and a limited range of motion (ROM) of the left shoulder. At clinical examination, neurovascular structures were intact. Conventional anteroposterior and axillary view radiographs showed an anatomical neck fracture with posterior dislocation of the humeral head (Figure 1A), confirmed by computed tomography (CT) scan with three-dimensional (3D) reconstruction (Figure 1B). 

**Figure 2:**
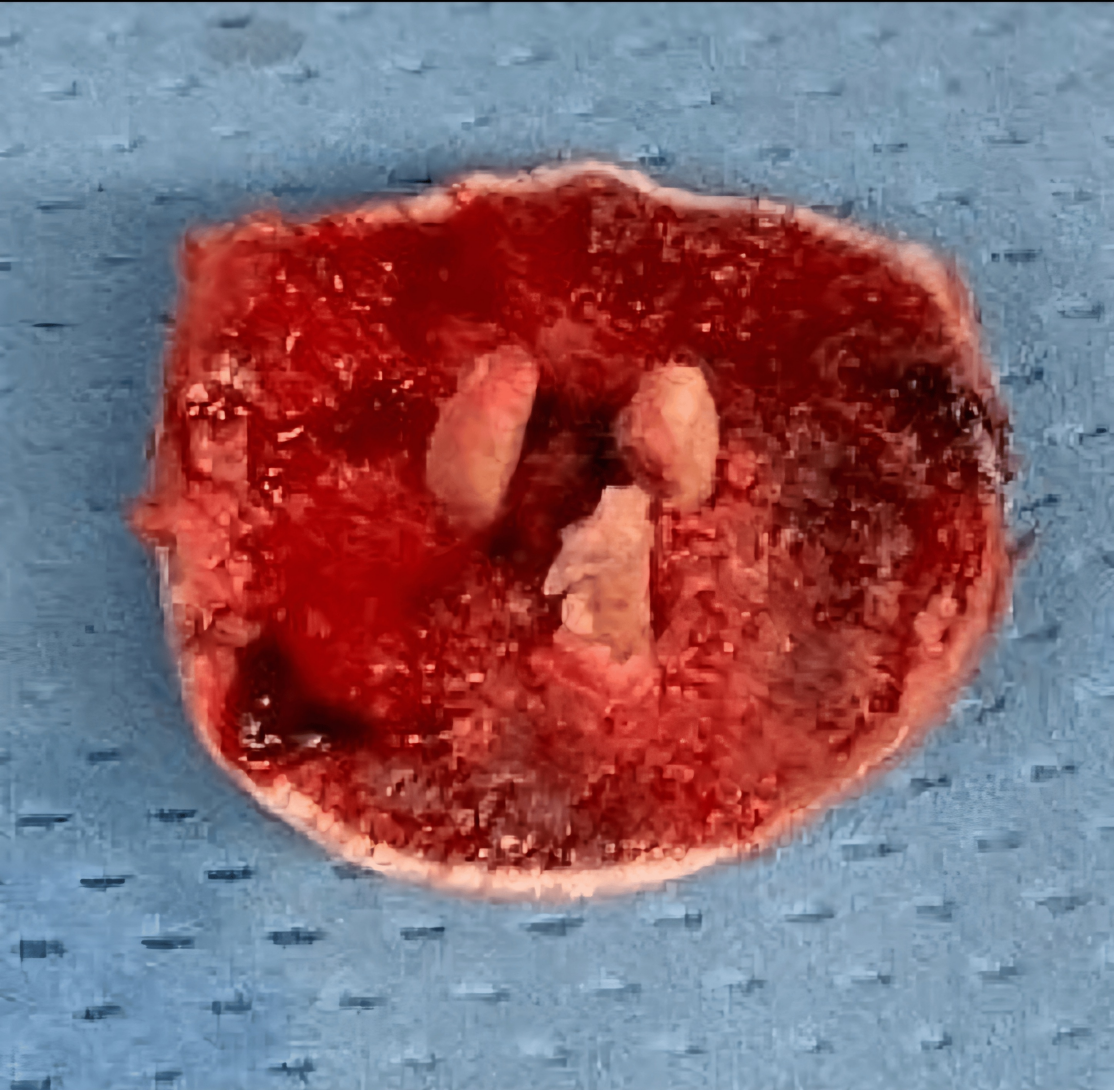
The implanted construct, made of humeral head and tibial grafts.

The fracture was classified as an 11C1 fracture according to Arbeitsgemeinschaft für Osteosynthesefragen/Orthopedic Trauma Association (AO/OTA) Classification [7] and a type II fracture according to Hertel’s Lego System [6]. After an unsuccessful attempt at reduction under anesthesia, the patient was admitted to the Trauma Unit, and, given his young age and the good general conditions, we planned ORIF with bioabsorbable screws and tibial autograft. A standard deltopectoral approach was performed with the patient in the beach chair position. The subscapularis tendon was incised 1 cm medial to the bicipital groove, leaving 10 mm laterally for reinsertion. The subscapularis tendon and the anterior capsule were meticulously split. The subscapularis tendon was prepared with #2 FiberWire sutures. The humeral head was free of soft tissue attachments and dislocated posteriorly, so it was removed. The humeral shaft and tuberosities were intact and still attached to the rotator cuff muscles. A tibial crest autograft of 2 x 1 cm was harvested from the left upper tibia; three small slices were obtained from the graft and implanted into the humeral head (Figure 2).

**Figure 3:**
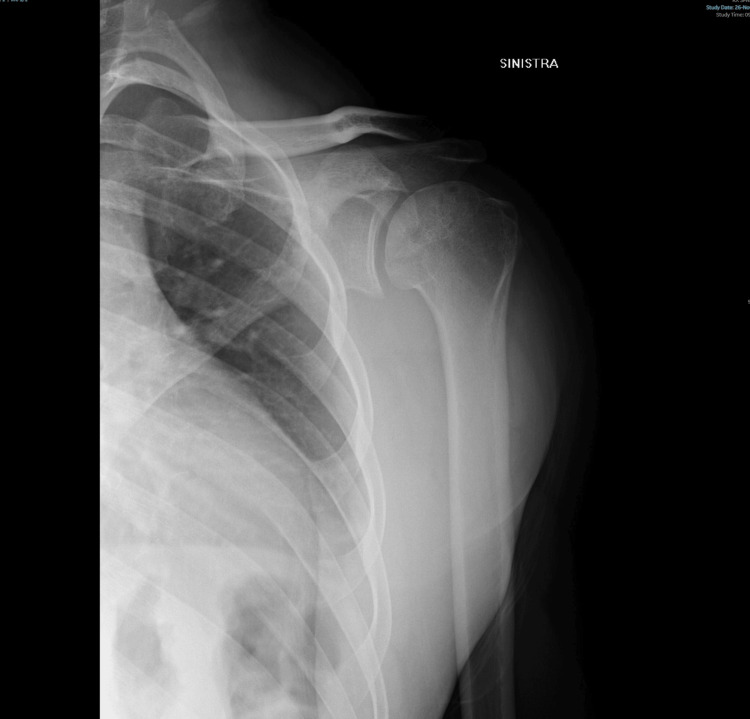
Immediate postoperative anteroposterior radiograph.

The construct was then re-implanted and fixed using three poly-L-lactic acid (PLLA) 2.7 x 26 mm Bio-Compression screws. The screws were inserted slightly convergent to each other, with an angle of 135° with respect to the diaphysis of the humerus, with the aim of increasing the stability of the construct. Anatomical reduction and full restoration of the articular surface with good stability were obtained. The post-operative radiograph is shown in Figure 3.

**Figure 4:**
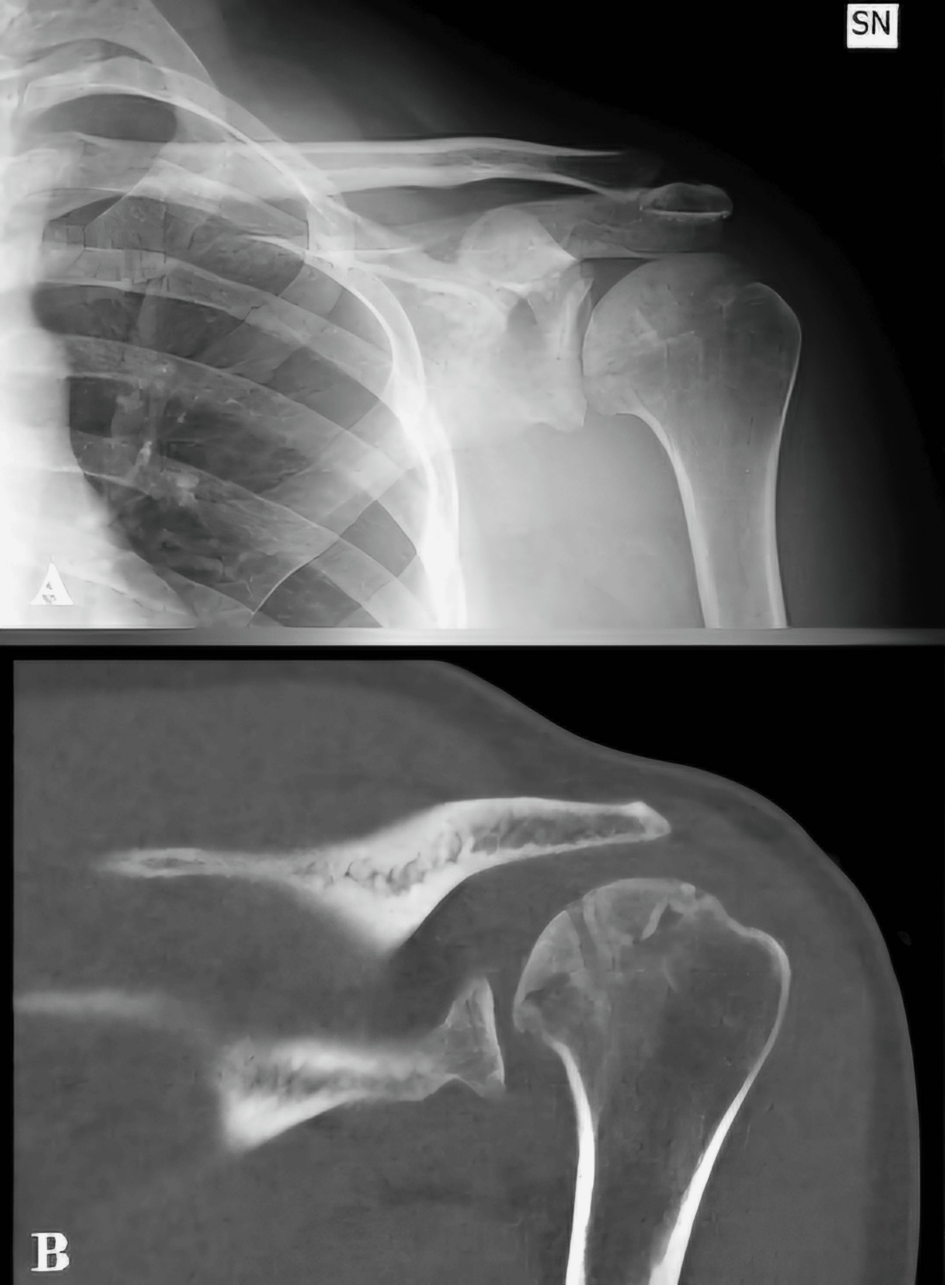
(A) Anteroposterior plain radiograph and (B) CT scan image in coronal plane at sixth-month follow-up.

A sling and swathe brace was applied after surgery in the operating room and maintained for four weeks. Two weeks after surgery, control sutures were removed, and pendulum Codman’s and passive-assisted exercises were started. At one month, clinical and radiographic evaluation was performed, initial signs of fracture and graft consolidation were seen, and active shoulder rehabilitation was prescribed. At the three-month follow-up, the patient did not report any limitations during daily activities, and a full ROM was restored. Radiograph showed complete integration of the graft, so muscle strengthening and stretching were also prescribed. At six months follow-up, conventional radiographs and a CT-scan were obtained to evaluate bone healing, showing complete bone consolidation without any sign of avascular necrosis of the humeral head (Figures 4A, 4B). At clinical examination, the patient presented a full ROM, with mild pain at maximum degrees of internal and external rotation of the shoulder. Clinical tests executed (jerk test, posterior load and shift test, posterior apprehension test, and shoulder posterior drawer test) resulted in negative findings.

Nine months after surgery, the patient was fully satisfied with a full return to pre-injury activities, including motorcycle driving and overhead sports. At the two-year follow-up, the patient regained a full range of motion, with optimal muscle strength and shoulder stability. He reported no difference between the operated and uninjured shoulder. Given the absence of symptoms and excellent function, the patient did not wish to undergo any further imaging. Three years after the injury, the patient is still asymptomatic.

## Discussion

The reported case describes the successful management of a two-part proximal humerus fracture dislocation in a young patient treated with bioabsorbable screws and tibial crest autograft. Proximal humerus fractures are unusual in young patients, in whom optimal management remains controversial [8]. The management of such fractures is challenging, overall due to the critical decision-making in selecting appropriate treatment [9]. When ORIF is the choice, surgeons face significant technical difficulties, particularly in achieving an anatomical reduction of the humeral head and associated fractures [10]. Despite meticulous anatomical restoration, a significant risk is represented by the compromised vasculature of the humeral head, which may lead to aseptic necrosis, observed in over 40% of cases [9,11].

This patient presented only two fragments, but there was a complete separation between the head and the shaft with unavoidable interruption of the anterior circumflex artery that provides the primary vascularization to this region [12]. The main debate in the surgical choice revolves around the risk of avascular necrosis of the humeral head [13]. Hemiarthroplasty and ORIF can produce comparable results [9], but in young adults, ORIF is usually preferred [14]. Hemiarthroplasty must be subjected to rigorous evaluation when considered a treatment strategy [15]. While hemiarthroplasty may be selected in cases where ORIF is challenging, its indication in young patients must be balanced against the potential benefits and risks [16]. Although reverse total shoulder arthroplasty is a viable option, it could lead to severe complications or hardware failure if not performed by expert surgeons [17]. So, ORIF may represent the initial treatment choice, but the possibility of requiring further revisions or alternative procedures, such as hemiarthroplasty or reverse total shoulder arthroplasty, highlights the complexity of managing these fractures in the younger population [18]. Therefore, this case represented a significant challenge because of the rare and complex posterior shoulder fracture-dislocation and the young age of the patient. In young patients, cautious surgical techniques are mandatory to achieve optimal outcomes and minimize long-term complications, satisfying the higher functional requests and expectations. So, hemiarthroplasty or reverse total shoulder arthroplasty was not selected as the treatment choice.

Gavaskar and Tummala, in their study, indicated that ORIF had satisfactory outcomes in simple head-splitting fractures, while complex fractures were correlated to higher rates of non-union, avascular necrosis, and lower shoulder functionality [19]. Douleh et al. reported a patient with a four-part proximal humerus fracture with subcoracoid dislocation and devascularization in whom the humeral head was explanted and then reimplanted and fixed with a plate and screws without any complication, such as osteonecrosis, at 12 months [20]. Hohmann et al., in their meta-analysis of surgical treatment for proximal humerus fractures in middle-aged and elderly patients, reported that, in terms of clinical outcomes, ROM, and complication rates, ORIF was superior to hemiarthroplasty and comparable to intramedullary nailing (IM). At the same time, reverse shoulder arthroplasty was superior to hemiarthroplasty but comparable to ORIF [6].

Despite concerns regarding potential compromised vasculature and aseptic necrosis, no radiographic signs of osteonecrosis were evident at the six-month follow-up. This implies that the chosen treatment approach may have diminished the risk of this event. The patient's successful recovery promises good long-term outcomes, and repeated monitoring is recommended to assess healing and functional progress. Avoiding humeral head osteonecrosis, our approach represents a feasible alternative for such complex fractures in selected young patients. If our novel approach fails and revision surgery is needed, or if shoulder osteoarthritis develops in the future, hemiarthroplasty or reverse shoulder arthroplasty could be performed.

## Conclusions

Open reduction and internal fixation using bioabsorbable screws and tibial crest autograft offers an alternative to standard ORIF and shoulder arthroplasty in young adults with proximal humerus fracture-dislocation. The approach successfully restored anatomical integrity and function without complications, highlighting its potential as a valuable treatment strategy in select cases. The current technique showed the potential to avoid humeral head osteonecrosis while preserving the option for future arthroplasty should failure occur. However, further studies are needed to assess the reproducibility and long-term outcomes of this approach.
